# Effect of low-frequency repetitive transcranial magnetic stimulation combined with motor imagery training on upper limb motor recovery and primary motor cortex activation in stroke patients: A randomized controlled trial

**DOI:** 10.1097/MD.0000000000046083

**Published:** 2025-11-21

**Authors:** Jong-Bae Choi, Seo-Won Yang, Ji-Su Park

**Affiliations:** a Department of Occupational Therapy, Chosun University, Gwangju, Republic of Korea; b Department of Occupational Therapy, Doowon Technical University, Gyeonggi-do, Republic of Korea; c Research Institute for Korean Medicine, Pusan National University, Yangsan, Republic of Korea.

**Keywords:** low-frequency repetitive transcranial magnetic stimulation, motor imagery training, primary motor cortex activation, stroke, upper limb function

## Abstract

**Background:**

We aimed to investigate the effects of low-frequency repetitive transcranial magnetic stimulation (LF-rTMS) combined with motor imagery training (MIT) on upper limb function recovery and primary motor cortex (M1) activation in patients with stroke.

**Methods:**

Forty-four patients with stroke were randomly assigned into 2 groups: experimental (both LF-rTMS combined with MIT, N = 22), and control (LF-rTMS, N = 22) groups. The treatment was performed for 20 minutes a day, 3 days a week, for 8 weeks. To evaluate upper limb function, the Fugl–Meyer assessment for upper extremity, wolf motor function test, and action research arm test were conducted. To evaluate M1 activation, motor-evoked potential amplitude was evaluated before and after conducting the study.

**Results:**

The results indicated that both groups showed significant changes across all evaluation items before and after the intervention. In the prepost and change comparisons, the experimental group demonstrated significantly greater changes in the FMA-UE, wolf motor function test, and action research arm test than the control group. Moreover, the combined intervention of LF-rTMS and MIT was effective in improving upper limb function and activating M1 in patients with severe stroke.

**Conclusion:**

We propose this intervention as a novel clinical intervention method for recovering upper limb function in patients with stroke.

## 1. Introduction

Patients with stroke generally exhibit hemiplegia on the contralateral side of the affected hemisphere and complex dysfunction in upper extremity movements.^[1]^ Upper limb hemiplegia is one of the most common disabilities after stroke, affecting >80% of patients in the acute phase and >40% of patients in the chronic phase.^[2]^ Upper extremity dysfunction affects a variety of activities of daily living and may limit participation in social activities.^[3]^ The recovery of upper extremity function following stroke remains incomplete and demonstrates a poor prognosis; therefore, treatment advances have been made in recent decades to restore upper extremity function in the early stages after stroke.^[4,5]^ Recently, studies have reported that a combined approach of various interventions is effective in restoring upper limb function compared to a single intervention.^[6]^ Several studies have shown that improvement in upper extremity function after stroke can be improved through noninvasive brain stimulation techniques combined with various clinical interventions.^[7–9]^ Repetitive transcranial magnetic stimulation (rTMS) is a noninvasive brain stimulation that can modulate cortical activity. Stroke can result to limitations in physical functions due to an imbalance in interhemispheric cortical inhibition. Accordingly, rTMS can reestablish interhemispheric balance by suppressively regulating the excitability of the intact hemisphere with low-frequency stimulation (LF) or upregulating damaged excitability with high-frequency stimulation (HF).^[9]^ LF-rTMS is performed based on the theory of transcallosal inhibition (TCI), which can be explained by a model of competition between cerebral hemispheres. In normal people, both cerebral hemispheres contribute to competing with or controlling the contralateral cerebral hemisphere, and this control is explained as interhemispheric inhibition through the corpus callosum.^[10,11]^ In a previous study, rTMS applied to the primary motor cortex (M1) of the cerebral cortex on the nondamaged side of patients with stroke activated M1 on the damaged side; this activation explained TCI, indicative of a disinhibition mechanism.^[12]^ LF-rTMS, which uses frequencies <1 Hz for suppressing neuronal excitability in the brain, modulates stroke-induced imbalanced interhemispheric interactions. A recent meta-analysis study reported the therapeutic effect of LF-rTMS for improving upper extremity function after stroke.^[13]^ Additionally, noninvasive neuromodulation combined with upper limb motor rehabilitation interventions can improve upper limb motor function following stroke.^[14,15]^ Studies have demonstrated that maximal control of the lesioned hemisphere is associated with improved upper extremity function.^[16,17]^ Zhang et al^[13]^ evaluated the therapeutic potential of LF rTMS for stroke-induced upper limb motor impairment and cortical plasticity. They found that the application of additional clinical interventions combined with LF-rTMS significantly promoted improvements in upper extremity function after stroke.^[13]^ Technologies that can be simultaneously applied with rTMS include motor imagery training (MIT), action observation, virtual reality, and neuromuscular functional electrical stimulation.^[18]^ Considering that patients with severe upper limb injuries in the acute phase after stroke may have limited active movement, complete lack of physical activity may limit their participation in various treatments requiring active movement. MIT, a noninvasive neuromodulation technique, has been proven effective in improving upper extremity function as an alternative intervention to increase the rehabilitation effect of patients recovering from severe stroke, particularly those with limited active movement.^[19,20]^ MIT is a cognitive activity in which patients mentally simulate specific movements without explicit movements.^[21]^ Thus, it can be applied to anyone, regardless of the patient’s upper limb motor function level. Previous studies have shown that MIT and motor execution share the same neural networks involved in motor function.^[22,23]^ These findings corroborated the idea that MIT can be employed to supplement physical movement activities where patients are unable to move.^[24]^ Moreover, it can be applied in patients with severe motor impairment.^[25]^ Several studies have shown that MIT exhibit similar characteristics with real-world behavior in terms of temporal regularities, programming rules, and biomechanical constraints.^[26,27]^ Kawakami et al^[28]^ investigated the cortical changes following MIT in patients with chronic stroke and reported positive plasticity changes during mental training for M1. Meanwhile, Mihara et al^[24]^ demonstrated that M1 activation can be improved in conjunction with MIT and potentially have a significant impact on the recovery of motor deficits in patients with stroke. Additionally, they found that changes in cortical activation were associated with recovery of hand function.^[24]^ Several studies have reported that this single intervention method is effective in improving upper limb function in patients with stroke, and thus it is necessary to confirm its effectiveness through research as a parallel intervention.

In this study, LF-rTMS aims to enhance the rehabilitative potential of the lesioned hemisphere by modulating cortical excitability based on neurophysiological mechanisms, while MIT is intended to activate higher-order motor areas through cognitive processes. Therefore, this study seeks to investigate the synergistic effects of the combined intervention compared to each intervention alone.

Considering that LF-rTMS and MIT hold no limitations as a combined intervention for improving upper limb function in patients with severe stroke, this study sought to maximize the recovery of motor function in patients by combining the 2 interventions. Currently, few studies have attempted to investigate the effects of LF-rTMS combined with MIT on upper limb function and M1 activation. Therefore, we aimed to investigate the effects of combined rTMS and MIT on improving upper limb motor function and activating M1 in patients with severe stroke hemiplegia. We hypothesized that this combined intervention would promote improvements in upper limb function after severe stroke.

## 2. Materials and methods

### 2.1. Study design

**Figure 1.**
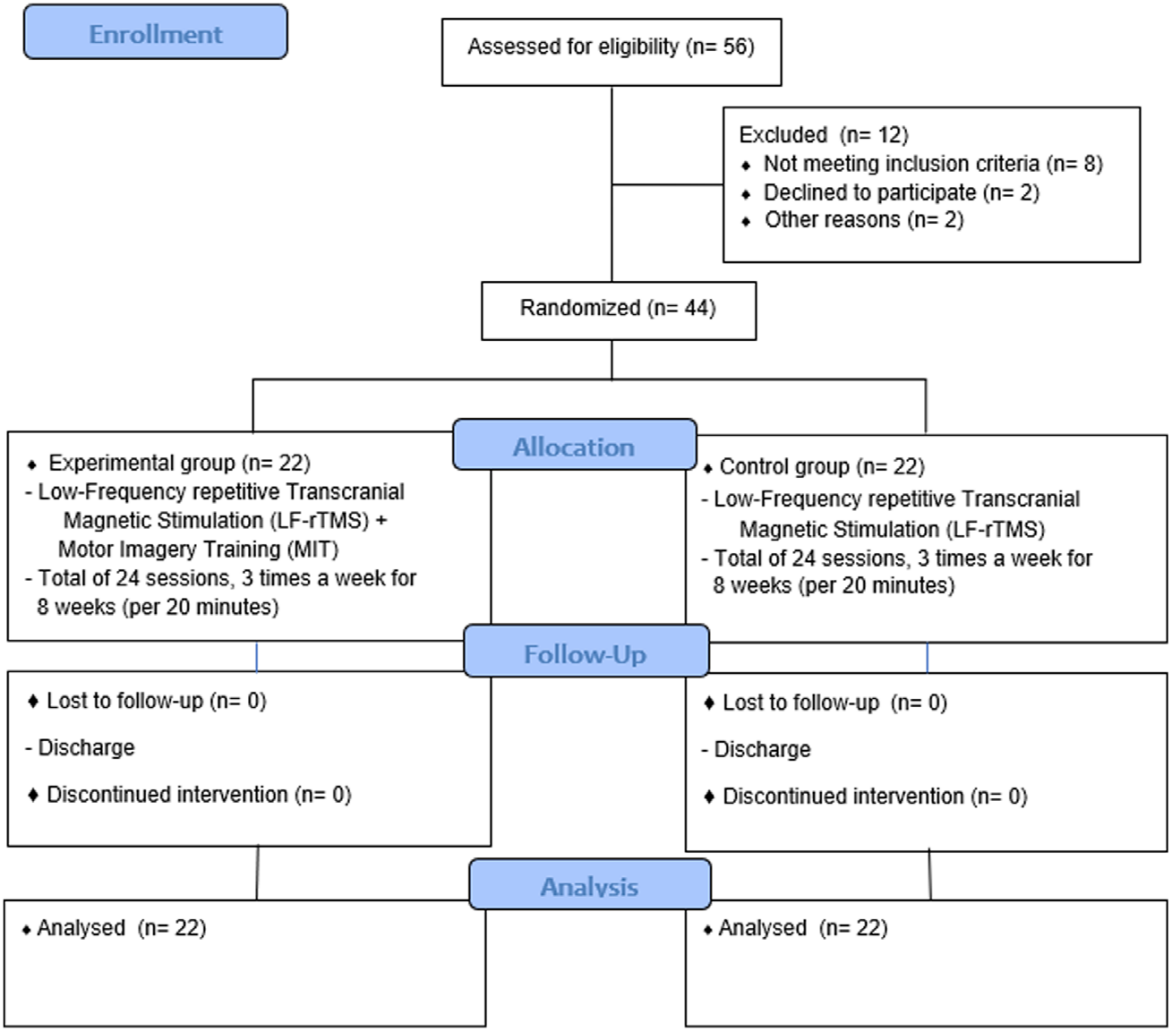
CONSORT flow diagram of participant recruitment. CONSORT = consolidated standards of reporting trials.

The participants include 44 subacute patients in the recovery stage within 6 months of stroke onset hospitalized at H Rehabilitation Hospital in Gyeonggi-do between January 2023 and June 2023. The sample size was set to 44 participants for the mean comparison (*T* test) of the 2 groups using the G-Power program 3.1, with a significance level of 0.05, a power of 0.8, and an effect size of 0.8.^[29]^ To minimize selection bias, 22 patients were randomly assigned to each of the experimental and control group using a computer random number table program (Fig. 1).

The criteria for patients with severe upper limb functional impairment were based on a previous study.^[30]^ Patients diagnosed with stroke and hemiplegia by a rehabilitation medicine doctor; those with subacute hemiparesis <6 months after stroke onset; those with an MMSE-K score of ≥24 who can understand and execute the instructions; those with a grade ≤3 (*F*) in the manual muscle test of the wrist extensor muscles; and those with severe upper extremity function impairment with an FMA-UE score of ≤19, were included. The exclusion criteria were as follows: presence of an artificial pacemaker; severe upper extremity pain on the paretic side (visual analog scale score ≥ 5); presence of metal in the skull; or history of pacemaker, intracardiac wire.

### 2.2. Ethical compliance

Patients who understood the purpose and procedures of the study and voluntarily agreed to participate were enrolled. Written informed consent was obtained from all participants. The study was conducted in accordance with the principles of the Declaration of Helsinki and approved by the Institutional Review Board of Chosun University (IRB No. 2-1041055-AB-N-01-2024-03). Furthermore, the study was registered with the Clinical Research Information Service of South Korea (CRIS No. KCT0009391).

### 2.3. Study procedure

This study was a 2-group experimental control study, and all progress and evaluations were conducted by 2 occupational therapists with >10 years of clinical experience. Outcome assessments were performed by independent evaluators who were blinded to group allocation to minimize detection bias. A total of 44 hospitalized patients were randomly assigned to either the experimental or control group using a computer-generated random number table. The randomization sequence was generated by an independent researcher who was not involved in participant enrollment or intervention. Allocation concealment was ensured using sequentially numbered, opaque, sealed envelopes (SNOSE), which were opened only after the participant consented and completed baseline assessments. The 2 groups received traditional rehabilitation treatment for 30 minutes a day, thrice a week, for 8 weeks. During the same period, the experimental group received LF-rTMS and M1, and the control group received LF-rTMS alone for a total of 20 minutes. To evaluate the improvement of upper limb function, Fugl–Meyer assessment upper extremity (FMA-UE), wolf motor function test (WMFT), and action research arm test (ARAT) were used. To evaluate M1 activation, motor-evoked potential (MEP) amplitude was measured using TMS.

### 2.4. Low-frequency repetitive transcranial magnetic stimulation (LF- rTMS)

**Figure 2.**
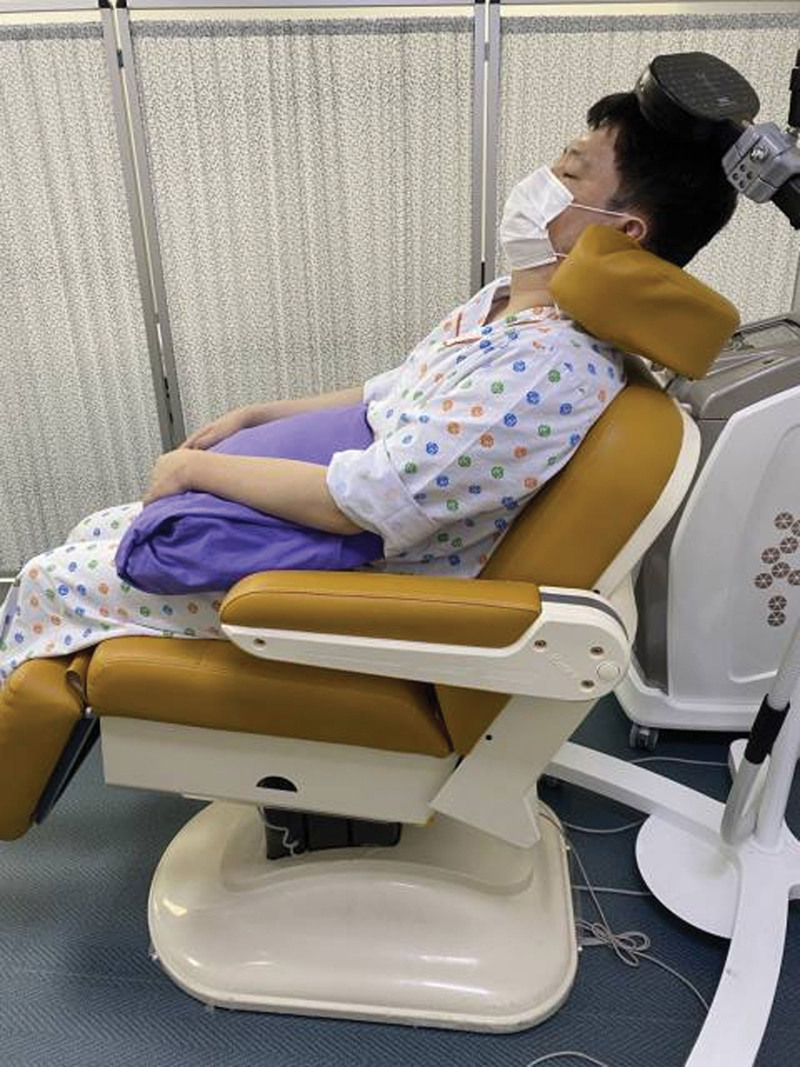
An actual photograph of a participant receiving low-frequency repetitive transcranial magnetic stimulation.

The LF-rTMS employed the ALTMS® (Remed, Republic of Korea), and the stimulation was performed using a 70 mm figure-8 coil. Participants were instructed to maintain a comfortable and relaxed posture in the device’s chair. Subsequently, their head was gently stabilized on the headrest, with both arms and elbow joints extended. The wrist joint was maintained in a neutral position, while the forearm was placed in a prone position, and the fingers lightly extended. To check the motor threshold value, the participants worn a hood to identify the stimulation location, and the coordinates are marked accordingly. The coordinates are drawn from the nasion to the inion, and a point is subsequently created by intersecting the mid sagittal and the inter aural line. Based on this line, it is created by crossing lines in a checkerboard shape with 1 cm intervals each. The coil stimulator is placed on the affected cerebral hemisphere at an angle of 45° from the center line (Fig. 2). MEP was measured using the first dorsal interosseous (FDI), which is involved in hand movement, as the target muscle. To check the location of M1 of the FDI muscle, the patient’s scalp was stimulated by slightly moving its position. The largest MEP value of the FDI muscle is determined as the motor cortex area of the muscle. The resting motor threshold is set as the minimum stimulation intensity at which MEPs of ≥50 μV are recorded in at least 5 out of 10 stimulations.^[31]^ The stimulation intensity was set to 90% of the resting motor threshold, with a stimulation frequency of 1 Hz, and the stimulation was performed for 20 minutes for a total of 1200 stimulations per session. Repetitive transcranial magnetic stimulation treatment was performed thrice a week for 8 weeks for a total of 24 sessions.^[32]^

### 2.5. Motor imagery training (MIT)

Based on previous study, the modified audio-based MIT consisted of 20-minutes sessions.^[33]^ In the case of the audio-based MIT, it was performed according to audio recordings in which participants were asked to imagine the movements of body joints of the upper limbs and movements of daily living activities. MIT was performed simultaneously while LF-rTMS were being performed. Prior to the MIT, the occupational therapist explained the content of the MIT training to all participants until they fully understood the training. The entire MIT course is divided into 3 parts. First, imagination preparation (1 minute): participants were encouraged to immerse themselves in the imagination state. Recorded instructions guided the participants to close their eyes, take deep breaths, enter a state of relaxation, and gradually fall into an imaginary state. Second (5 minutes): the participants were instructed to imagine moving the joints of the hemiplegic upper extremity (e.g., shoulder flexion, elbow/wrist flexion and extension, hand grasp and release, 3 jaw pinch, tip pinch). Third, imagination of movements of daily living activities (15 minutes): participants were asked to imagine daily living activities (e.g., washing hands, using chopsticks, drinking water, brushing teeth, using a cell phone, getting dressed, writing, using a computer keyboard).

### 2.6. Outcome measures

FMA-UE is a method of evaluating motor function on the paralyzed side of patients with stroke, and this study evaluated only the upper limb items of FMA-UE. The FMA-UE consists of 33 items and evaluates upper limb functional ability, which comprise 18 items from the shoulder, elbow, and forearm; 5 items from the wrist; 7 items from the hand and fingers; and 3 items from coordination ability. It is divided into a 3-point scale and can be scored from 0 to 2 points. The score is measured based on the participant’s level of performance. A score of 0, 1, and 2 indicates no performance, partial performance, and complete performance, respectively. The total score of upper extremity function consists of 66 points. FMA-UE was confirmed to have very high reliability, with inter-rater and intra-rater reliability of 0.97.^[34]^

WMFT is a measurement tool that quantifies upper extremity motor ability through functional task performance. It is a complex evaluation tool that evaluates the participant’s task performance time, functional ability, and muscle strength. It is a 6-point scale ranging from 0 (unable to perform with the upper extremity on the paretic side) to 5 (performs normal movements using the upper extremity on the paretic side). It consists of 17 tasks ranging from simple to complex movements. The highest score is 75 points, and the higher the score, the more independent and normal functioning can be interpreted. The inter-rater reliability of the function score of this tool is .88, and the inter-rater reliability of the performance time is .97.^[35]^

ARAT was used to evaluate the upper extremity executive function of patients with stroke. The ability to hold and carry several objects of various sizes, and weights was checked. ARAT consist of 19 items, including grasping (6 items), grip (4 items), pinching (6 items), and gross movements (3 items). The scale is a 4-point scale from 0 to 3, with higher scores indicating a higher level of performance. The total score is 57 points: 0 points if unable to perform, 1 point if partially performed, 2 points if the task was completed but took a long time or showed difficulty, and 3 points if the task was performed normally and completely. All tasks during the evaluation were performed using only the affected hand. The intra-examiner reliability of the ARA test is 0.99 and test–retest reliability is 0.98.^[36,37]^

In this study, MEP amplitudes were measured using the ALTMS® (Remed, Republic of Korea). MEP is an objective electrodiagnostic evaluation tool that induces specific muscle responses through transcranial magnetic stimulation of the cerebral cortex. For magnetic stimulation, the international electroencephalograph 10 to 20 recording method was applied, and the central part of the coil stimulator was placed at the Cz position. The FDI muscle is located in the motor cortex at a 45° angle from the center line and moves gradually to determine the point at which the maximum response occurs. The maximum magnetic field strength was 2.0 T and the stimulation time was 0.1 ms.^[38]^ Stimulation intensity was gradually increased from 80% to 100%, and stimulation occurred multiple times. EMG values were measured using the belly-tendon method by attaching a silver-silver chloride electrode to the FDI muscle on the affected side and a ground electrode to the arm. Resting motor threshold was defined as the minimum stimulation intensity at which MEPs > 50 μV were recorded at least 5 times during 10 stimulations. The amplitude of MEPs was determined by measuring the amplitude 12 times after 120% stimulation.^[39]^ Peak-to-peak amplitudes of evoked MEPs from contralateral target muscles were obtained. Electromyography values were obtained using mobile KEY POINT®.NET software, and the signal was amplified at 100 ms/div and subsequently filtered from 2 Hz to 10 kHz.

### 2.7. Statistical analysis

The data collected in this study were analyzed using the SPSS version 22.0 (SPSS Inc., Chicago) program. To analyze the patients general characteristics, including demographic and clinical variables, homogeneity was tested using frequency analysis of descriptive statistics and the chi-squared test. Paired *t* test was used to determine the average change before and after intervention within the experimental and control groups. Independent samples *t* test was conducted to determine the difference between the 2 groups. Additionally, an independent samples *t* test was performed to compare the average change before and after the experiment between the 2 groups. The significance level of all statistical data was set at *a* = 0.05.

## 3. Results

### 3.1. Participants characteristics

The general characteristics of the study participants are listed in Table 1. A homogeneity test was conducted on the demographic and clinical variables of the participants between the 2 groups, and no significant differences were found (Table 1).

**Table 1:** General characteristics of participants.

Characteristics	Experimental group (n = 22)	Control group (n = 22)	*X*²/*t*	*P*-value
Age (yr), mean ± SD	65.41 ± 5.84	66.92 ± 6.35	−1.364	.867
Sex (male/female)	11/11	9/13	0.578	.664
Type of stroke (hemorrhage/infarction)	14/8	12/10	−1.478	.592
Side of stroke (right/left)	12/10	11/11	−0.215	.907
Time since onset of stroke months, mean ± SD	5.94 ± 1.65	6.14 ± 1.53	−0.367	.717

SD = standard deviation.

### 3.2. Pre- and post-intervention comparison of upper extremity function in the experimental and control groups

In the pre- and post-intervention comparison within the 2 groups, both groups showed significant changes in the upper limb function evaluations FMA-UE, WMFT, and ARAT, as well as the MEP amplitude evaluation to compare M1 activation. In the before-and-after comparison between the 2 groups, the experimental group showed greater significant changes than the control group in FMA-UE, WMFT, and ARAT, which evaluate upper limb function, and in the MEP amplitude evaluation to compare M1 activation (Table 2).

**Table 2:** Preintervention and postintervention comparison of upper extremity function in the experimental and control groups.

	Experimental group (n = 22)			Control group (n = 22)			Between groups (*P*-values)
	Before treatment	After treatment	*P*-value	Before treatment	After treatment	*P*-value	
FMA-UE	11.65 (4.72)	22.68 (5.31)	<.001*	10.48 (5.62)	17.36 (6.91)	<.001*	.015†
WMFT	10.37 (5.46)	18.24 (6.19)	.002*	9.63 (4.17)	13.25 (4.73)	<.001*	.024†
ARAT	8.26 (3.83)	16.75 (4.68)	<.001*	7.92 (3.13)	10.48 (4.58)	.024*	.007†
MEP amplitude (μV)	110.46 (47.61)	281.96 (107.57)	<.001*	98.46 (40.78)	179.51 (92.41)	.002*	.000†

Values are represented as mean (SD).

ARAT = Action Research Arm Test, FMA-UE = Fugl–Meyer Assessment for Upper Extremity, MEP = motor-evoked potential, WMFT = Wolf Motor Function Test.

* *P* < .05 by paired *t*-tests.

† *P* < .05 by independent *t*-tests.

### 3.3. Between-group comparison of pre- to post-intervention change scores

**Figure 3.**
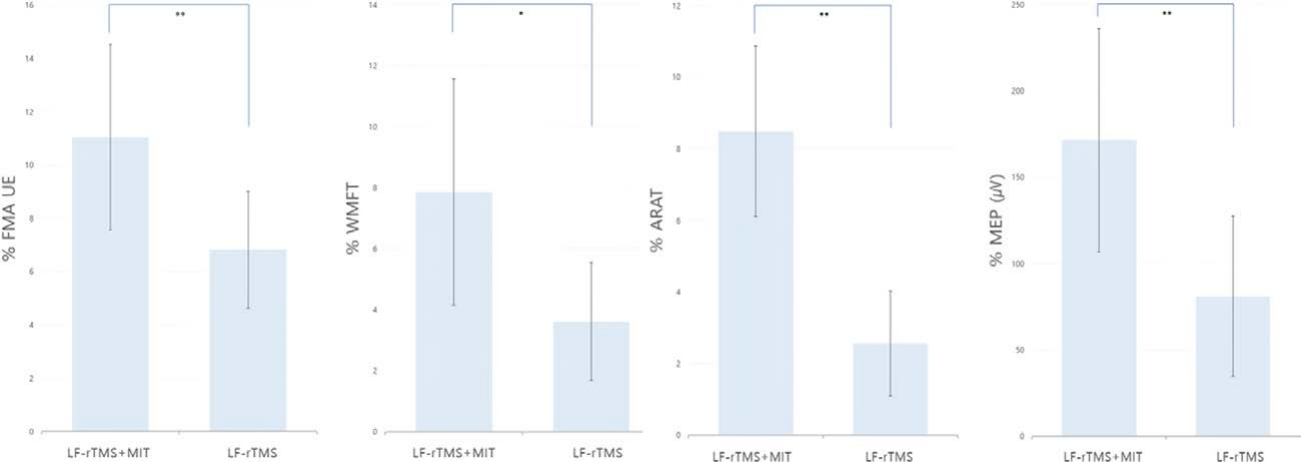
Comparison of pre- and post-intervention changes between the experimental and control groups.

The experimental group exhibited a significantly greater change than that in the control group in the upper limb function evaluation, including FMA-UE, WMFT, and ARAT, as well as in the MEP amplitude evaluation to compare M1 activation (Table 3, Fig 3).

**Table 3:** Between-group comparison of preintervention to postintervention change scores.

	Experimental group	Control group		*P*-value
FMA-UE	11.04 (3.47)		6.8 (2.18)	.012*
WMFT	7.87 (3.71)		3.62 (1.93)	.021*
ARAT	8.49 (2.39)		2.56 (1.47)	.001*
MEP amplitude (μV)	171.5 (64.51)		81.05 (46.45)	.008*

Values are represented as mean (SD).

ARAT = Action Research Arm Test, FMA UE = Fugl–Meyer Assessment for Upper Extremity, MEP = motor-evoked potential, WMFT = Wolf Motor Function Test.

* *P* < .05 by independent *t*-tests.

## 4. Discussion

Recovery of upper extremity motor function following stroke is important. Studies showed that functional recovery occurs several years after stroke due to cortical neuroplasticity.^[40]^ However, up to 50% of patients with stroke continue to experience persistent and severe upper limb paralysis even after receiving rehabilitation treatment. Consequently, patients with severe upper limb functional impairment experience difficulty in participating in general upper limb care.^[41]^ Accordingly, studies have been conducted on several new single and parallel arbitration methods. Recent reports showed that combining other interventions rather than single interventions is more effective in improving upper extremity function in patients with stroke due to a positive synergistic effect.^[18,33]^ Among them, LF-rTMS is a safe and noninvasive method of stimulating the cerebral cortex, which does not only improve the brain’s ability to relearn task-specific functions, but also increase the rehabilitation effect by regulating corticomotor excitability.^[42]^ Concomitantly, MIT is effective in improving upper limb function through a cognitive rehearsal process in patients with stroke with severe lower limb function and limited active movement.^[21]^ Therefore, in this study, we investigated the effects of LF-rTMS combined with MIT on upper limb functional recovery and M1 activation in patients with severe stroke.

A prepost comparison study within the 2 groups to evaluate the recovery of upper limb function showed that both the experimental group of LF-rTMS combined with MIT and the control group of only LF-rTMS showed significant changes in the evaluation of FMA-UE, WMFT, and ARAT. LF-rTMS alone can have a significant impact on improving upper extremity function in patients with stroke, and several previous studies corroborate this finding. Kim et al^[43]^ conducted a study comparing 3 groups of patients with severe stroke: LF-rTMS, HF-rTMS, and do-nothing groups. Significant changes in FMA-UE were observed in both rTMS groups compared to the control group.^[43]^ Niimi et al^[44]^ also reported significant improvement in WMFT through LF-rTMS intervention in patients with stroke.

When comparing the extent of change in the upper extremity function between the 2 groups, the experimental group showed greater significant changes in the evaluation of FMA-UE, WMFT, and ARAT. Comparing the change in FMA-UE (6.8 ± 2.18) of the control and experimental (11.04 ± 3.47) group, an increase in the minimum clinically importance difference score of 9 to 10 was observed. In another study, the MCID criteria of WMFT (1.0–1.2 points) and ARAT (5.7 points) showed significant changes in the experimental group. Accordingly, the amount of change in the experimental group can be presented as evidence for greater improvement in upper limb function.^[45–47]^ Pan et al^[33]^ reported significant improvements in FMA-UE and WMFT in an initial study of LF-rTMS + MI concurrent intervention, supporting the results of this study. However, this was an early study that did not evaluate the short 4-week intervention and cerebral cortex activation. Accordingly, the finding that the LF-rTMS + MI parallel intervention of this study, which complemented the limitations of previous studies, was effective in improving upper limb function is more meaningful. The M1 in the premotor area, which is activated during actual movement of the upper limb, was activated through MIT.^[24,26]^ In addition, it demonstrated complex synergistic effect in improving upper limb function by directly activating M1 through parallel intervention of LF-rTMS.

In the evaluation of MEP amplitude within both groups to assess M1 activation, both groups showed significant changes. Several previous studies have reported changes in cerebral cortex neuroplasticity through significant improvement in MEP amplitude via the LF-rTMS in patients with stroke, which supports the findings of this study. Du et al^[48]^ reported M1 activation through functional magnetic resonance imaging (fMRI) evaluation with LF-rTMS intervention. Regarding the change in M1 activation between the 2 groups, the experimental group showed a greater significant change in MEP amplitude evaluation than the control group. This provided evidence that the combined LF-rTMS + MIT intervention could elicit greater changes in M1 activation than the LF-rTMS intervention alone. As a result, activation of the M1 area, which is responsible for motor function commands in the central nervous system, can trigger action potential signal transmission to muscles responsible for upper limb functions in the peripheral nervous system. Increased MEP amplitude reflects enhanced corticospinal excitability, which is often associated with improved motor performance and recovery prognosis. This may indicate better motor planning and readiness of M1 for voluntary movement, particularly relevant in severely impaired stroke patients.^[49]^ This can lead to substantial changes in upper limb movement. The above upper limb function evaluation has been reported to exhibit significant changes. Previous studies have underscored the activation of corticospinal pathways through event-related desynchronization of LF-rTMS + MIT parallel intervention in healthy participants, which supports the results of this study.^[50,51]^ However, as those study targeted healthy adults, the findings of the present study is more meaningful since it reports significant changes in M1 activation in severe patients with stroke. LF-rTMA is a TCI mechanism that activates the lesioned M1 area by suppressing neural activation in the area opposite the lesion. Furthermore, the additional intervention for direct activation of the M1 area through MIT, likely accelerated the activation of the M1 area on the damaged side, leading to a positive synergy effect. This study has several limitations, one of which is that the participants were recruited from a single institution, the sample size was small, and the intervention period was limited to 8 weeks, thereby restricting the generalizability of the findings. In future studies, it will be necessary to recruit a larger number of participants across multiple institutions and conduct long-term research to provide more objective and reliable findings. Moreover, since the participants were patients in the subacute stage of stroke, they may be affected by natural brain function recovery. The lack of an objective fMRI neurophysiological evaluation tool to assess changes in cerebral cortex activation, suggests the need for further research in the future.

## 5. Conclusions

We aimed to investigate the effects of LF-rTMS + MIT combined intervention on upper limb function and M1 activation in patients with stroke. The results indicated that the combined LF-rTMS + MIT intervention exhibited a more significant effect on upper limb function and M1 activation in severe patients with stroke compared to that when LF-rTMS is employed alone. Therefore, we propose this as a new concurrent treatment intervention for patients with severe stroke and provide scientific evidence.

## Author contributions

**Conceptualization:** Seo-Won Yang.

**Data curation:** Seo-Won Yang, Ji-Su Park.

**Funding acquisition:** Jong-Bae Choi.

**Investigation:** Jong-Bae Choi, Seo-Won Yang.

**Methodology:** Jong-Bae Choi, Seo-Won Yang.

**Project administration:** Seo-Won Yang, Ji-Su Park.

**Software:** Ji-Su Park.

**Supervision:** Seo-Won Yang.

**Validation:** Seo-Won Yang.

**Writing – original draft:** Jong-Bae Choi, Seo-Won Yang, Ji-Su Park.

**Writing – review & editing:** Jong-Bae Choi.
